# Effect of Door-to-Door Screening and Awareness Generation Activities in the Catchment Areas of Vision Centers on Service Use: Protocol for a Randomized Experimental Study

**DOI:** 10.2196/31951

**Published:** 2021-11-04

**Authors:** Shalinder Sabherwal, Anand Chinnakaran, Ishaana Sood, Gaurav K Garg, Birendra P Singh, Rajan Shukla, Priya A Reddy, Suzanne Gilbert, Ken Bassett, Gudlavalleti V S Murthy, Operational Research Capacity Building Study Group

**Affiliations:** 1 Department of Community Ophthalmology and Public Health Research Dr Shroff’s Charity Eye Hospital New Delhi India; 2 Department of Projects and Marketing Dr Shroff’s Charity Eye Hospital New Delhi India; 3 Department of Optometry Dr Shroff’s Charity Eye Hospital New Delhi India; 4 Indian Institute of Public Health Hyderabad India; 5 School of Medicine, Dentistry and Biomedical Sciences Queen’s University Belfast United Kingdom; 6 Seva Foundation Berkeley, CA United States; 7 University of British Columbia Vancouver, BC Canada; 8 See Authors' Contributions

**Keywords:** study protocol, randomized intervention study, vision centers, door-to-door screening, cost-effectiveness, sustainability, screening, awareness, vision, eye, utilization, usage, India, rural, intervention, engagement, scalability

## Abstract

**Background:**

A vision center (VC) is a significant eye care service model to strengthen primary eye care services. VCs have been set up at the block level, covering a population of 150,000-250,000 in rural areas in North India. Inadequate use by rural communities is a major challenge to sustainability of these VCs. This not only reduces the community’s vision improvement potential but also impacts self-sustainability and limits expansion of services in rural areas. The current literature reports a lack of awareness regarding eye diseases and the need for care, social stigmas, low priority being given to eye problems, prevailing gender discrimination, cost, and dependence on caregivers as factors preventing the use of primary eye care.

**Objective:**

Our organization is planning an awareness-cum-engagement intervention—door-to-door basic eye checkup and visual acuity screening in VCs coverage areas—to connect with the community and improve the rational use of VCs.

**Methods:**

In this randomized, parallel-group experimental study, we will select 2 VCs each for the intervention arm and the control arm from among poor, low-performing VCs (ie, walk-in of ≤10 patients/day) in our 2 operational regions (Vrindavan, Mathura District, and Mohammadi, Kheri District) of Uttar Pradesh. Intervention will include door-to-door screening and awareness generation in 8-12 villages surrounding the VCs, and control VCs will follow existing practices of awareness generation through community activities and health talks. Data will be collected from each VC for 4 months of intervention. Primary outcomes will be an increase in the number of walk-in patients, spectacle advise and uptake, referral and uptake for cataract and specialty surgery, and operational expenses. Secondary outcomes will be uptake of refraction correction and referrals for cataract and other eye conditions. Differences in the number of walk-in patients, referrals, uptake of services, and cost involved will be analyzed.

**Results:**

Background work involved planning of interventions and selection of VCs has been completed. Participant recruitment has begun and is currently in progress.

**Conclusions:**

Through this study, we will analyze whether our door-to-door intervention is effective in increasing the number of visits to a VC and, thus, overall sustainability. We will also study the cost-effectiveness of this intervention to recommend its scalability.

**Trial Registration:**

ClinicalTrials.gov NCT04800718; https://clinicaltrials.gov/ct2/show/NCT04800718

**International Registered Report Identifier (IRRID):**

DERR1-10.2196/31951

## Introduction

Primary care is the cornerstone of the global health system and is rooted in the 1978 Declaration of Alma Ata [1], encompassing disease prevention and the equitable distribution of health care [2]. Derivative to this, the Global Action Plan for Universal Eye Health [3] emphasizes the importance of providing basic eye care to all individuals, and the communities they constitute, at affordable rates [4]. An application of the bottom-up approach, primary eye care is an integral part of comprehensive eye care: promoting eye health, increasing accessibility, and linking individuals and the community to health care systems [5-8].

In India, primary eye care is delivered through two main mechanisms:

- Transient screening camp-scheduled, community-based activities that screen patients, provide glasses to those requiring them at the camp itself, and transport those needing surgery to the base hospital [9].
- Permanent facilities: Vision centers (VCs) with catchment areas of roughly 50,000 people, mostly located in rural areas and urban slums and accessible by public transport [10,11]. They refract, diagnose, and treat minor eye conditions and refer cases needing further care to their nearest base hospital [12].

Globally, awareness regarding eye health [13], need-based demand [13], financial issues and cost [13,14], and poor communication from providers [14] are the major barriers to primary eye care use. The literature on barriers to primary eye care in India is limited but points to a lack of knowledge about eye diseases, detrimental social stigmas, low priority being accorded to eye problems, gender discrimination, unaffordability, a lack of perceived need, and immobility and dependence on escorts [15-18]. These barriers to the access and use of services have the potential to affect the overall operational sustainability of the VCs, affected in large part by the number of walk-in patients [19].

Our organization is a network of eye care delivery mechanisms based on the pyramidal model [10] and spread across North India. Currently, 36 VCs (9 urban and 27 rural) are under operation, raising awareness; providing refraction, recognition, and referral services to their catchment population; and increasing contact of those in need of services with doctors through teleophthalmology. For the majority of people, these primary eye care centers are the first point of contact when accessing or attempting to access eye care services. Moreover, gender differences have been established in the use of VC services, with the proportion of women among the walk-in patients being higher compared to men in urban VCs and lower in rural ones [12].

Thus, generating awareness, developing trust, and improving access to these VCs, amongst the entire catchment population they service, is essential not only for the overall sustainability of these centers but also to bring more and more people under the ambit of primary care delivery. Previously, a door-to-door screening model was posited to eliminate avoidable blindness [15]. This research protocol aims to study the effect of an intervention combining door-to-door screening with regular awareness activities in the catchment population on service use at VCs. The overall cost-effectiveness of such an intervention will also be analyzed.

## Methods

### Study Design and Process

This study is a randomized, parallel-group experimental study in which we selected four VCs, two each in the intervention and control arms (one each from a particular operational area).

Our organization has six secondary centers, of which four are located in the state of Uttar Pradesh, namely Meerut, Mathura, Saharanpur, and Kheri. These regions have a total of 23 VCs operating in rural and semiurban areas, together serving around 1 million people. Of these four secondary centers, two were selected (Vrindavan in Mathura District and Mohammadi in Kheri District) for this study based on feasibility and the demographic profile of their catchment population.

**Figure 1:**
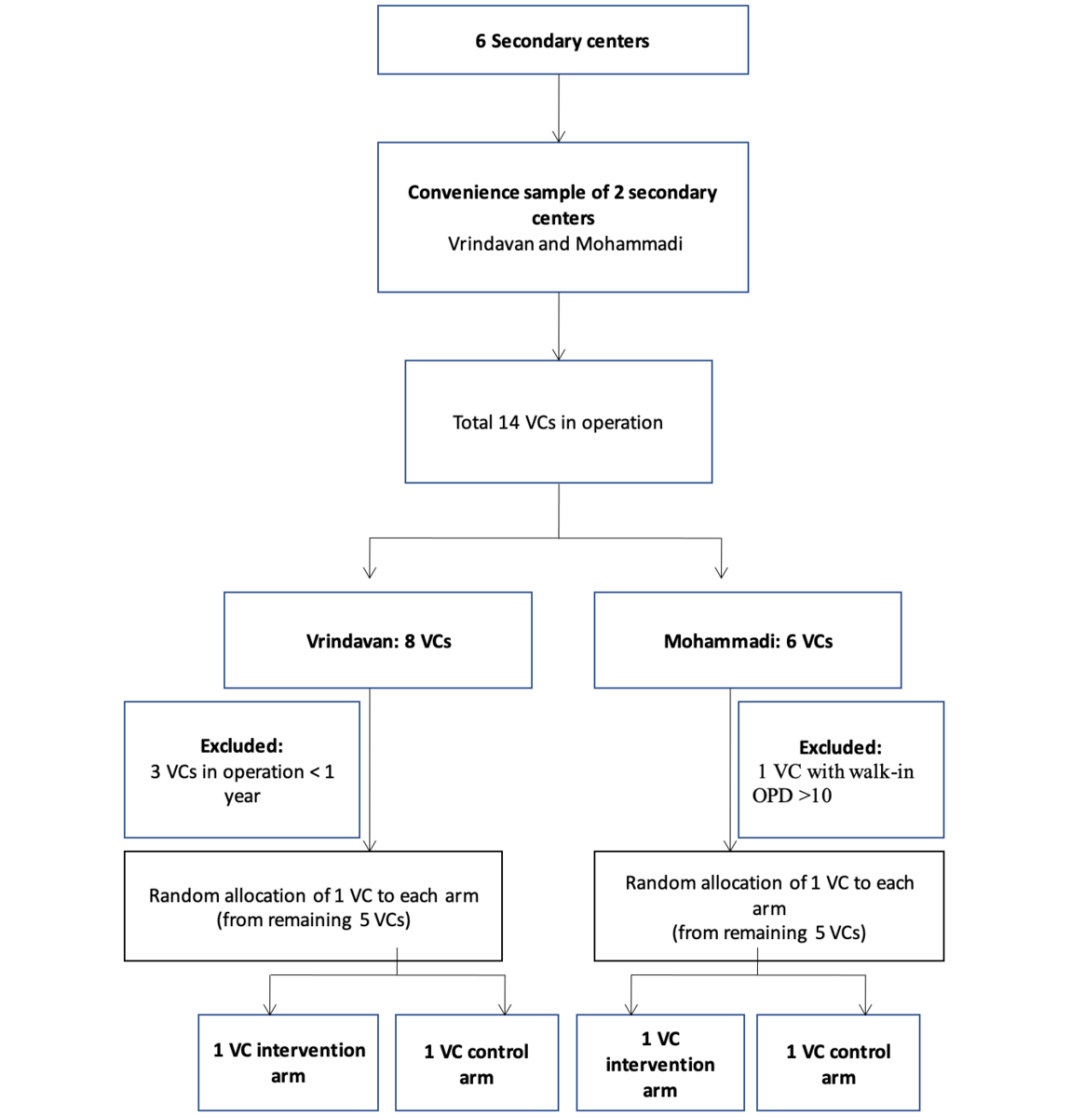
Selection process used to identify the study vision centers (VCs). OPD: outpatient department.

The Vrindavan region has eight VCs delivering eye care services in its semiurban areas, while the Mohammadi region has six VCs (five rural and one semiurban). Most of these VCs have been operational for over 3 years. However, data from the previous year indicated that 80% of the VCs are suboptimal in their performance. The VCs performing suboptimally were listed. From a total of 10 VCs (5 in each region, meeting the inclusion/exclusion criteria), 2 were randomly selected from each region using the RAND function in Microsoft Excel (ie, 1 each for the control and the intervention arm). The process is illustrated in detail in Figure 1.

The VCs in the two blocks of Mathura District are located at Chhata and Raya, while the two VCs in the two blocks of Kheri District were located at Mitauli and Pashgaon. Due to the nature of the study, it was not possible to mask the field staff to the intervention.

### Inclusion/Exclusion Criteria

The regional team gathered detailed information regarding all VCs operating in the Vrindavan and Mohammadi regions. VCs were included in the study based on the following inclusion criteria:

- Low performance (walk-in OPD ≤ 10 per day)
- Duration of operation > 1 year
- Presence of one VC in each arm from selected VCs

VCs with walk-in outpatient department (OPD) numbers greater than 10 per day and those in operation for less than 1 year were excluded.

### Study Setting

Mathura District has a population of about 2.5 million, 70% of whom are resident in rural areas [20]. Kheri District has a population of around 4 million, of which 88% reside in rural areas [21].

Per our existing data, around 75% of the patients visiting our VCs in these 2 districts reside within 10 km of our VCs. The Chhata block, in Mathura, has 81 villages, with around 30 of those being within 10 km of the VC. These 30 villages have a combined population of around 70,000. In contrast, the Raya block has 124 villages, with around 90 of these being within 10 km of the VC, having a combined population of around 130,000.

In Kheri District, the Mitauli block has 138 villages, of which 75 are within 10 km of the VC. The combined population of these villages is around 111,000. The Pashgaon block has 230 villages, with around 85 of these being within 10 km of the VC, having a combined population of around 110,000.

### Sample Size

The average OPD attendance at the intervention centers is 7 per day at present. We expect to achieve 14 after the intervention, failing which, the intervention will not be considered a success. Therefore, the primary objective of the statistical analysis would be to estimate the average attendance, postintervention, with extreme precision, which will lead us to assess whether we have been able to achieve the target (14 per day, on average). We set the confidence interval (CI) to ±1 for the postintervention sample mean (the narrowest-possible CI in this case). To check whether at least 20 per day on average has been achieved, we expect a sample mean of at least 21 (ie, the CI will be 20-22). The number 20 has been determined based on a feasibility study conducted to project VC sustainability. We assume that the probability of the CI is 95% and the daily OPD attendance has a Poisson distribution. Thus, we expect that the postintervention daily attendance will have a Poisson distribution with a mean of at least 21. This implies that the variance of the distribution will also equal 21 (or more), and we want to estimate the mean with a 95% CI of ±1. This requires a minimum sample size of 81 days.

We used the following formula to calculate the sample size:

Sample size = (1.96^2^/*d*^2^) × Variance of the distribution

where 1.96 is the 97.5 percentile point of the standard normal distribution and d is the length of the CI (on one side of the estimate). If d = 1 and variance = 21, we obtain sample size = 81. We set a target of 20 per day in the pre-CODIV-19 time, but due to the pandemic, we revised our desired target to 14 per day to consider the intervention a success. We persisted with the additional days in the sample size (instead of 58 days for a target of 14) to be able to estimate up to 14 per day OPD attendance with precision.

### Intervention Arm

Our VC team includes a technical person (a trained vision technician) and a community health worker (a VC attendant). Although the vision technician is responsible for patient examination, the VC attendant assists the vision technician and carries out community engagement activities. In addition, each region has a VC coordinator to supervise all VCs in that region. The intervention will include door-to-door screening and awareness generation in 8-12 villages in the catchment area of the intervention VC. We will leave the villages adjacent to the VCs and instead approach a mix of near and distant villages within our catchment area. A list of surrounding villages (within 10 km) will be prepared by the VC coordinator and the VC attendant. The VC coordinator will meet each village leader to take permission for the door-to-door intervention survey to be carried out in the village. After having received the necessary permissions, a priority list of survey villages will be prepared to initiate the intervention.

The VC attendant will be trained to use the Peek acuity application [22] for measuring visual acuity and using the data collection software Taraka on Android platforms. They will also be trained to use the developed information, education, and communication (IEC) material. In the intervention villages, the VC attendant will go from door to door. During the screening, if any house is locked or family members are not available, the VC attendant will attempt to contact those missing at least three times. The VC attendant will explain the intervention and obtain verbal consent for participation in the survey. Household or family members who are unwilling or not interested to participate in the survey will also be recorded separately.

After obtaining verbal consent for the screening, the VC attendant will communicate regarding the need for eye care in general and share the IEC material. For each family member above 5 years of age, the visual acuity of each eye will be measured and recorded using the PEEK acuity application on a smartphone. Demographic data, ocular complaints, and information regarding any previous eye checkup will also be recorded in the Android application.

Any person with a visual acuity of <6/12 (cutoff) or other eye issues will be counseled and referred for a comprehensive examination to the VC. A referral slip will be provided to the patient when referred, mentioning the reason for referral. Referred patients’ records will be accessible to the vision technician (optometrist) through the software. Patients reporting to the VC for a comprehensive checkup and treatment, due to the door-to-door intervention, will be recorded using vision center management software (VCMS). Any patient requiring surgical treatment or further care will be referred to the respective secondary center. Free cataract services will be provided to patients unable to afford the same. Follow-up of referred patients will be performed by the coordinators in the field.

### Control Arm

The control arm VC will continue its routine awareness activities and health talk sessions in the community. The VC attendant will prepare a monthly activity plan and organize activities in the surrounding 8-10 villages. Persons with eye issues will be recorded and referred to the VC for further evaluation and treatment. Patients reporting to the VC will be registered in the VCMS. For surgical intervention or further care, patients will be referred to the respective secondary center. Follow-up of referred patients will be performed by the coordinators in the field.

A comparison of the activities of the community health workers in the two arms is summarized in Table 1. The activities will be the same in both the arms and will also be standardized in the same manner; only their mode and reach will be different.

**Table 1:** Comparison of activities of community health workers in the two study arms.

Serial number	Activity	Intervention arm	Control arm
1	Meeting with key stakeholders	Yes	Yes
2	Health talk sessions in community Refer patients to VC	No	Yes
3	Awareness activity through IEC distribution	Yes (door-to-door)	Yes (cluster meeting in village during visits)
4	Permission for door-to-door survey intervention	Yes	No
5	Door-to-door screening Refer patients to VC	Yes	No

### Project Timelines

The study period will be 12 months, of which 2 months will be spent preparing the study intervention and obtaining approvals, 3 months will be needed for preintervention work (ie, training the team, field preparation, finalizing the data collection format, and IEC development), and 4 months for the intervention and data collection; after data collection, the remaining 3 months will be used for data analysis and writing.

### Data Collection, Management, and Analysis

#### Data Collection and Variables

We will collect both electronic and manual data for both study arms. In the intervention arm, field-level data (door-to-door surveys) will be captured through software, while field-level activity in the control arm will be manually recorded in the activity register. VC-level data will be extracted from the VCMS, which will contain data for both control and intervention VCs. In both arms, programmatic data will be collected, which will include data of the villages screened, door-to-door screening, walk-in OPD visits, those reporting after referrals from the field, and spectacles advised and their uptake, as well as referrals for cataract, specialty, and surgical follow-up (Figure 1 and Multimedia Appendix 1). Cost data will be collected for direct, indirect, and opportunity costs, such as rent, human resources, overheads, and community activities (Multimedia Appendix 2). We will also collect data for revenue from the OPD, spectacles, and surgeries done.

Most of the data for analysis will be directly extracted from the existing software at the VCs. The rest of the data pertaining to the cost will be entered, collected, and monitored as part of regular processes in the field. This will make the data collection process streamlined and integrated into the regular operations. Although the costs incurred in running any program may vary for different providers, we feel that the detailed checklists will help in disaggregating that data for use by different service providers.

#### Quality Assurance

There will be three sources of data in this research. The data from the door-to-door screening will be collected using a customized Android application, the data of patients visiting VCs will be captured through the VCMS, and the additional data pertaining to activities from the control VCs and the visits of various members of the staff will be collected in the registers.

Checks and balances have been built into the software to ensure completion of data collection. A comprehensive checklist has been prepared to standardize the manual data collection. Random visits will be made periodically to the field to monitor screening, awareness generation activities, and data collection. Data collected during the day will be uploaded to the cloud server at least once at the end of the day, and that would be available for review. Thus, the quality of data will be ensured by the clearly defined roles of the team members involved in the intervention, appropriate resource allocation, and regular meetings with the team members. A regular review process will be followed to maintain quality assurance of the collected data, and at least 10% of the collected data will be cross-checked/verified by field supervisors. Surgery-related data of the patients referred from VCs would be extracted from the electronic medical records of the secondary hospital. Data will be collated monthly as part of routine program monitoring and independently audited. The composition of the data-monitoring committee is provided as Multimedia Appendix 3.

No adverse events for the screener or the participants undergoing screening are anticipated, as services being provisioned are per standard hospital protocols and no experimental treatment is being given. Any complications in this scenario will be reported and dealt with per standard hospital policies.

#### Data Analysis

The collected data will further be tabulated and analyzed by each study arm: distance of the village, age, gender, eye issues, visual acuity, compliance with treatment (medicine, surgery, glasses), and revenue and expenditure of VCs. The difference from baseline in the number of walk-in patients, referrals, uptake of services, and costs involved in intervention will be analyzed. The Z test for proportions will be performed to compare the change in walk-in patients between the two arms. *P*<0.05 will be considered statistically significant. Subgroup analysis with respect to age and gender will also be carried out.

#### Cost-Effectiveness Analysis

Cost-effectiveness analysis and incremental cost-effectiveness analysis will be performed, and the incremental cost for every additional beneficiary attending the VC will be calculated. To calculate the increase in the number of patients, the average number of patients visiting per day during the same months in the previous year will be subtracted from the average in the study period. A change in the control VCs, if any, will be further deducted from this before using this as the denominator for calculating cost-effectiveness.

### Outcomes

The primary outcome for this study will be an increase in the number of walk-in patients at the VCs from baseline (7-8 walk-in patients to 14 per day after the intervention period of 4 months). The secondary outcomes will be uptake of spectacles and uptake of surgery among those advised. If the intervention proves effective in terms of the number of people visiting the VCs, cost-effectiveness will also be a secondary outcome.

### Ethical Considerations

This study was approved by the institutional review board of Dr. Shroff’s Charity Eye Hospital (IRB/2020/APR/54), has been registered as a clinical trial (NCT04800718) [23], and will follow the tenets laid out in the Declaration of Helsinki. Protocol amendments will be shared with all relevant parties via email, and approval will be sought again.

Data will be encrypted and kept confidential. These confidential data will be anonymized, and personal data will only be visible to those responsible for implementation. The final data set will only be accessible to the research team. Trial results will be disseminated via publication.

## Results

Background work involved in planning the interventions and selecting VCs has been completed. Participant recruitment has begun and is currently in progress. We estimate the primary completion date (ie, the date on which participant enrollment ends) to be November 30, 2021, and the study completion date to be December 30, 2021.

## Discussion

### Importance of Principle Findings

To the best of our knowledge, there is no previous study assessing the impact of door-

to door intervention on the sustainability of VCs. VCs are evolving as an important model for primary eye care [7,10,19]. Any such model needs to be sustainable for it to be universally adopted. Uptake of glasses and uptake of surgery by patients are the major contributors to the sustainability of these VCs [24]. Both these parameters are dependent on the number of patients visiting the VC, and that will be assessed in our study.

### Addressing the Barriers to Uptake of Services

In their study describing barriers to the uptake of eye care services among the rural population, Marmamula et al [17] reported a lack of felt need as the most important person-related barriers. Thus, when designing our intervention package, we have included awareness generation as one of the key components. Other barriers detected in that population are the absence of someone to accompany, lack of accessibility, and affordability. Taking the preliminary screening to people’s doors in our intervention should manage, to some extent, the barriers to accessibility and the absence of an accompanist. We have also made the first examination at the VCs, free of cost for those reporting after a preliminary screening.

### Cost-Effectiveness of the Model

In addition to evaluating the effect on the number of patients visiting the VCs, our study will provide evidence for the cost-effectiveness of such an intervention. Although community engagement has been established as an important element of primary care [1], the evidence for the cost-effectiveness of a door-to-door screening model will help in decision making regarding the scalability of such an intervention.

### Generalizability of the Results

In India, like in many low-to-middle-income countries, the majority of the population resides in rural areas [25]. With an unequal distribution of doctors, including ophthalmologists, in rural locations [26], the need for primary care is greater there. All the VCs included in our study belong to such locations; thus, the learning can be used in other similar settings.

### Limitations

Although we randomly selected the VCs from our two operational regions, the fact that we operate only in North India can be one limitation of our study. We had planned this study before the COVID-19 pandemic, and even after reasonable delay due to the unrelenting nature of the pandemic, we plan to start this study during the ongoing pandemic. Due to this, the target for the number of patients visiting the VCs has reduced. Although the conditions may not be near normal during data collection, we do not anticipate any difference in the way in which the intervention and control VCs would be affected by the prevalent conditions. Due to the nature of the intervention, it is not possible to mask the personnel on ground, and this may bring in some short-term behavior change, which may not be sustained. Another limitation of our study would be the short duration of data collection following the intervention. To analyze the long-term impact of the intervention, another study will be planned subsequently in case the results of this study show a positive impact.

### Conclusion

We believe our results will provide evidence for the impact of the door-to-door screening model of community engagement, on VC sustainability. The cost-effectiveness analysis would help the community care organizations like us to decide the feasibility and scalability of such an intervention.

## Appendix

Multimedia Appendix 1 Data to be collected for the two study arms.

Multimedia Appendix 2 Data to be collected for calculating cost.

Multimedia Appendix 3 Data-monitoring committee.

## Abbreviations

CI: confidence interval
IEC: information, education, and communication
OPD: outpatient department
VC: vision center
VCMS: vision center management software

## References

[1] World Health Organization WHO Called to Return to the Declaration of Alma-Ata.

[2] Starfield B, Shi L, Macinko J (2005). Contribution of primary care to health systems and health. Milbank Q.

[3] World Health Organization Universal Eye Health: A Global Action Plan 2014?2019.

[4] Khan MA, Soni M, Khan MD (1998). Development of primary eye care as an integrated part of comprehensive health care. Community Eye Health.

[5] Murthy G, Raman U (2009). Perspectives on primary eye care. Community Eye Health.

[6] Gilbert C (1998). The importance of primary eye care. Community Eye Health.

[7] Misra V, Vashist P, Malhotra S, Gupta SK (2015). Models for primary eye care services in India. Indian J Community Med.

[8] World Health Organization (1997). Strategies for the Prevention of Blindness in National Programmes.

[9] Community Outreach Initiatives for High Quality, Large Volume, Sustainable Cataract Surgery Programmes. Aravind Eye Hospitals, Postgraduate Institute of Ophthalmology Lions Aravind Institute of Community Ophthalmology and Seva Foundation.

[10] Rao G, Khanna R, Athota S, Rajshekar V, Rani P (2012). Integrated model of primary and secondary eye care for underserved rural areas: the L V Prasad Eye Institute experience. Indian J Ophthalmol.

[11] World Health Organization Blindness and Vision Impairment.

[12] Sabherwal S, Sood I, Garg G, DasGupta S, Nagappan S, Reddy P, Bassett K Gender-Inequity in Eyecare: Variation by Service Level and Location in North India. IndianJPublic HealthRes Dev.

[13] Hayden C, Trudinger D, Niblett V, Hurrell D, Donohoe S, Richardson I, Applebee E Access to Primary and Secondary Eye Care.

[14] Holley C, Lee P (2010). Primary care provider views of the current referral-to-eye-care process: focus group results. Invest Ophthalmol Vis Sci.

[15] Bhoosnurmath K (2017). Hospital-based community eye health programme: a model for elimination of avoidable blindness on a sustainable basis. Community Eye Health.

[16] Kovai V, Krishnaiah S, Shamanna B, Thomas R, Rao G (2007). Barriers to accessing eye care services among visually impaired populations in rural Andhra Pradesh, South India. Indian J Ophthalmol.

[17] Marmamula S, Khanna RC, Shekhar K, Rao GN (2014). A population-based cross-sectional study of barriers to uptake of eye care services in South India: the Rapid Assessment of Visual Impairment (RAVI) project. BMJ Open.

[18] Dandona R, Dandona L, Naduvilath T, McCarty C, Rao G (2000). Utilisation of eyecare services in an urban population in southern India: the Andhra Pradesh eye disease study. Br J Ophthalmol.

[19] Khanna RC, Sabherwal S, Sil A, Gowth M, Dole K, Kuyyadiyil S, Chase H (2020). Primary eye care in India: the vision center model. Indian J Ophthalmol.

[20] Census 2011 Mathura District.

[21] Census 2011 Kheri District.

[22] Bastawrous A, Rono HK, Livingstone IAT, Weiss HA, Jordan S, Kuper H, Burton MJ (2015). Development and validation of a smartphone-based visual acuity test (Peek acuity) for clinical practice and community-based fieldwork. JAMA Ophthalmol.

[23] National Library of Medicine (U.S.) (2021). Increasing Vision Center Service Utilization. Identifier NCT04800718.

[24] Seva Sustainability.

[25] Census 2011 Population Census 2011.

[26] Thomas R, Paul P, Muliyil J (2003). Glaucoma in India. J Glaucoma.

