# Correction: Effect of Door-to-Door Screening and Awareness Generation Activities in the Catchment Areas of Vision Centers on Service Use: Protocol for a Randomized Experimental Study

**DOI:** 10.2196/35824

**Published:** 2022-01-18

**Authors:** Shalinder Sabherwal, Anand Chinnakaran, Ishaana Sood, Gaurav K Garg, Birendra P Singh, Rajan Shukla, Priya A Reddy, Suzanne Gilbert, Ken Bassett, Gudlavalleti V S Murthy, Operational Research Capacity Building Study Group

**Affiliations:** 1 Department of Community Ophthalmology and Public Health Research Dr Shroff’s Charity Eye Hospital New Delhi India; 2 Department of Projects and Marketing Dr Shroff’s Charity Eye Hospital New Delhi India; 3 Department of Optometry Dr Shroff’s Charity Eye Hospital New Delhi India; 4 Indian Institute of Public Health Hyderabad India; 5 School of Medicine, Dentistry and Biomedical Sciences Queen’s University Belfast United Kingdom; 6 Seva Foundation Berkeley, CA United States; 7 University of British Columbia Vancouver, BC Canada; 8 See Authors' Contributions

In “Effect of Door-to-Door Screening and Awareness Generation Activities in the Catchment Areas of Vision Centers on Service Use: Protocol for a Randomized Experimental Study” (JMIR Res Protoc 2021;10(11):e31951) one error was noted.

The following text was originally published in the Acknowledgments section of the paper. This text has now been moved instead to the Authors’ Contributions section:

The Operational Research Capacity Building Group consists of the following authors, who contributed equally to the paper: Gudlavalleti VS Murthy, Rajan Shukla, Samiksha Singh, Shailaja Tetali, Suresh K Rathi, Hemant Mahajan, Melissa G Lewis, Hira Pant, Tripura Batchu, Anirudh G Gaurang, Suzanne Gilbert, Ken Bassett, Priya A Reddy, Parami Dhakhwa, Ram P Kandel, Kuldeep Singh, and Prasanna Sharma.

Accordingly, the affiliation for the group author Operational Research Capacity Building Group has been changed from:

See Acknowledgments

to:

See Authors’ Contributions

The corrections will appear in the online version of the paper on the JMIR Publications website on January 18, 2022, with the publication of this correction notice. Because this was made after submission to PubMed, PubMed Central, and other full-text repositories, the corrected article has also been resubmitted to those repositories.

