# Supplementary material for: Effect of Door-to-Door Screening and Awareness Generation Activities in the Catchment Areas of Vision Centers on Service Use: Protocol for a Randomized Experimental Study
Source: JMIR Res Protoc. 2021 Nov 4;10(11):e31951. doi: 10.2196/31951 (PMC8603175; doi:10.2196/31951)
Supplement: Multimedia Appendix 1 [file resprot_v10i11e31951_app1.docx]

| **Serial Number** | **Data Collection Type** | **Data Collection Source** | **Variables** | **Intervention Arm** | **Control Arm** |
| --- | --- | --- | --- | --- | --- |
| 1. | VC Profile | VC Profile Format (Manual) | - Duration of existence of VC - Other eye care providers in the region | Yes | Yes |
| 2. | Village Information | Village Mapping Manual | 1. Population of village 2. Distance from VC 3. Transport Medium to commute to VC 4. Literacy percentage | Yes | Yes |
| 3. | Awareness Activity | Awareness Activity Register | Awareness about vision and eye care (Proxy) | Yes | Yes |
| 4. | **Intervention**  Door-to-door intervention  (only for Intervention VC) | Taraka Software (Screening and referred)  VCMS (Reported at VC) | 1. Member Screened, age and Gender 2. Age of those referred 3. Gender of those referred 4. VA of those referred 5. Nature of problem acute/ chronic (to be noted in the field?) 6. Age of those reported at VC 7. Gender of those reported at VC 8. VA of those reported at VC 9. Age and Gender those not reported 10. Diagnosis those reported at VC 11. Spectacle Uptake 12. Cataract surgery done 13. Specialty surgery done | Yes | No |
| 5. | VC MIS | VCMS | 1. Age and gender of Walk in OPD / Day 2. Age and gender of Spectacle Advised 3. Age and gender of Cataract Advised 4. Age and gender of Specialty Advised | Yes | Yes |
| 6. | Uptake of Surgery | EMR | 1. Age and gender of Cataract done 2. Age and gender of Specialty done | Yes | Yes |
| 7. | Activity Report | Activity Register | 1. Numbers of referrals reporting from activity/month 2. Non reporting numbers | No | Yes |
| 8. | Revenue and Expenditure | EMR &  VC MIS | 1. Revenue from Surgeries (Paid / subsidized / free) 2. Revenue of VC 3. Operational expenditure of VC | Yes | Yes |
| 9 | Time allocation for intervention | Door to door MIS and Field travel register | 1. Total Hours spend in Field by attendant 2. Total Hours spend in Field by Coordinator 3. Total Hours spend in Field by VC Lead | Yes | Yes |
