# Supplementary material for: Effect of Door-to-Door Screening and Awareness Generation Activities in the Catchment Areas of Vision Centers on Service Use: Protocol for a Randomized Experimental Study
Source: JMIR Res Protoc. 2021 Nov 4;10(11):e31951. doi: 10.2196/31951 (PMC8603175; doi:10.2196/31951)
Supplement: Multimedia Appendix 2 [file resprot_v10i11e31951_app2.docx]

| **Cost Outlay Items** | **Descriptions of cost** | **Source of Collection** | **Documents /Means of verification** | **Periodicity of Collection** | **Basis of Cost allocation to intervention** |
| --- | --- | --- | --- | --- | --- |
| **Direct Cost** | | | | | |
| **Software Development** | Development & Procurement | Finance | Software | Once | Total cost proportionate to using software in number of VC and number of lifespans of software |
| **Smart Phone for screening** | Smart Phone | Research Master Data Collection | Check List | Once | Proportionate cost of using mobile lifespan of tablet |
| **Training** | Software orientation, IEC, Travel, Boarding, Other | Training Team | Training Report | Periodically | Total Cost |
| **IEC Material - Banner, Leaflet** | Designing and printing | Stores / Finance | Indent / Material Issues slips | Monthly | Total Cost |
| **Human Resource Involved in intervention** | Salary of VT, Attendant, Vision Centre Coordinator, Sr team | Human Resource | Attendance Record | Monthly | Proportionate HR cost based on time contributed for intervention (apart from the regular activity) |
| **Travelling** | Traveling expenses of VT, Attendant, Vision Centre Coordinator, Sr team | Administration / Finance | Traveling record | Monthly | Proportionate traveling cost based on travel done for intervention (apart from the regular activity) |
| **Stationary & Printing** | Format, referral slips, registers etc. procurement | Stores / Finance | Indent / Material Issues slips | Monthly | Total Cost |
| **Uniform for survey** | Uniform design and procurement | Stores / Finance | Indent / Material Issues slips | Monthly | Total Cost |
| **Indirect Cost** | | | | | |
| **Monitoring** | HR involved | Research Master Data Collection | Meeting Log Records | Monthly | Proportionate to time allocation of monitoring for intervention (apart from the regular program monitoring) |
| **Consumables** | Consumable material procurement | Stores / Finance | Indent / Material Issues slips | Monthly | Total Cost |
| **Centrally Administrative Expenses** | Expenses of utilities used | Research Master Data Collection | Resource Allocation Records | Monthly | Proportionate cost based on time / days contributed for intervention (apart from the regular activity) |
| **Computer** | Procurement & Maintenance | Finance / Research Master Data Collection | Resource Allocation Records | Monthly | Proportionate cost based on time / days contributed for intervention (apart from the regular activity) |
| **Printer** | Procurement & Maintenance | Finance / Research Master Data Collection | Resource Allocation Records | Monthly | Proportionate cost based on time / days contributed for intervention (apart from the regular activity) |
