# Supplementary material for: Effect of Door-to-Door Screening and Awareness Generation Activities in the Catchment Areas of Vision Centers on Service Use: Protocol for a Randomized Experimental Study
Source: JMIR Res Protoc. 2021 Nov 4;10(11):e31951. doi: 10.2196/31951 (PMC8603175; doi:10.2196/31951)

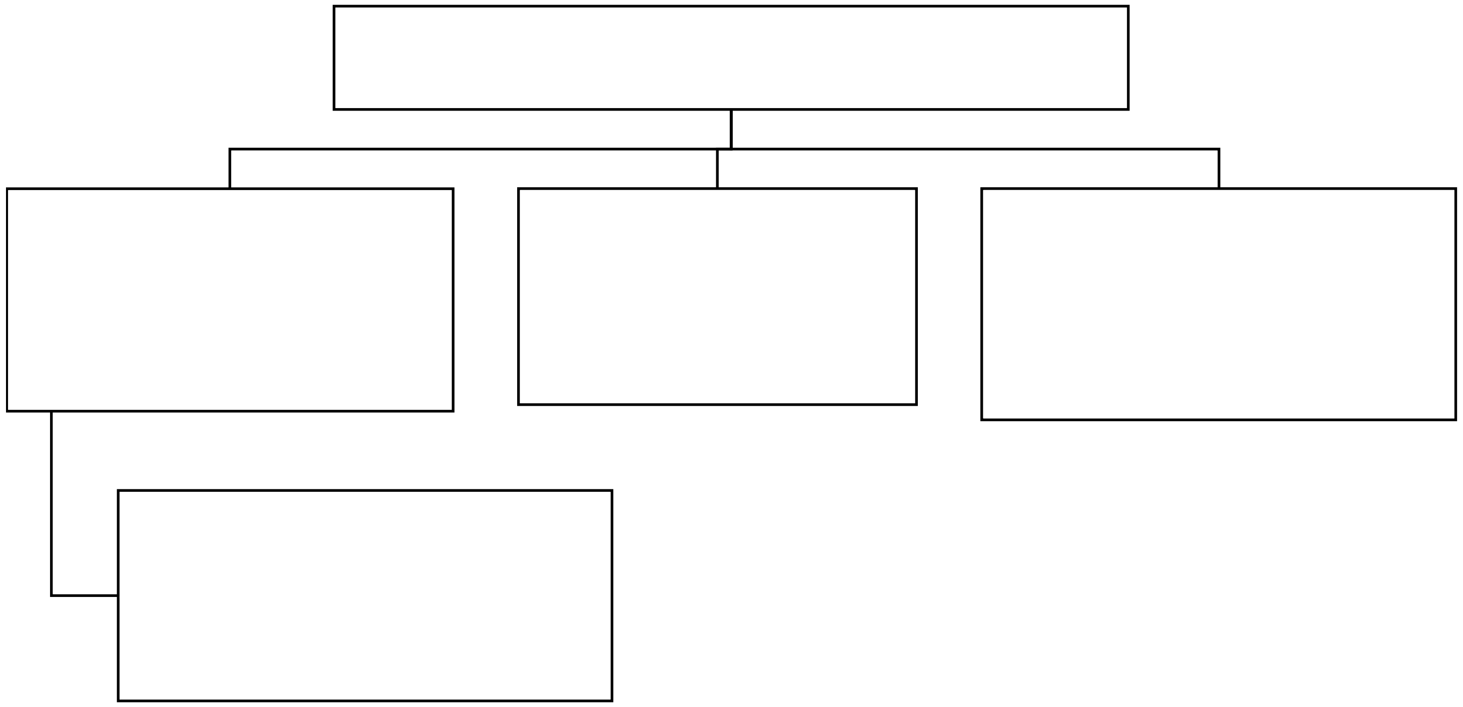

Supplement: Supplementary file 3 [file resprot_v10i11e31951_app3.docx]
